# Long-Term Efficacy of Adalimumab in Patients With Intestinal Behcet’s Disease: Eight Consecutive Cases

**DOI:** 10.14740/jocmr2477w

**Published:** 2016-02-27

**Authors:** Satoshi Tanida, Tsutomu Mizoshita, Hirotada Nishie, Keiji Ozeki, Takahito Katano, Takaya Shimura, Eiji Kubota, Hiromi Kataoka, Takeshi Kamiya, Takashi Joh

**Affiliations:** a Department of Gastroenterology and Metabolism, Nagoya City University Graduate School of Medical Sciences, Nagoya, Japan; b These authors contributed equally to this work.

**Keywords:** Ileocecal ulcer, Anti-TNF-α antibodies, Endoscopic assessment, Clinical remission

## Abstract

The long-term efficacy and safety of adalimumab (ADA) for the treatment of intestinal Behcet’s disease (BD) in the clinical setting have not been evaluated previously. This retrospective study evaluated the 52-week efficacy of ADA in BD patients. A total of eight patients who were refractory to conventional therapy were given ADA (160/80/40 mg every other week). Marked improvement (MI) was achieved by 10 weeks in five patients (62.5%), and by 52 weeks in six patients (75%). In addition, complete remission was obtained in two patients (25%) at both 10 and 52 weeks. Improvement of global gastrointestinal (GI) symptoms to score 0 was observed in three patients (37.5%) at 10 weeks and four patients (50%) at 52 weeks. Moreover, improvement of endoscopic assessment to score 0 was also seen in four patients (50%) at both 10 and 52 weeks. No adverse events were observed in any patients during the 52 weeks. In conclusion, ADA offers an effective, well-tolerated treatment for intestinal BD in patients who are refractory to conventional therapy.

## Introduction

Behcet’s disease (BD) is a chronic inflammatory disease characterized by repeated periods of remission and deterioration of oral and genital ulcers, along with ocular or skin involvements [1]. These manifestations are caused by systemic vasculitis. Intestinal BD, which is associated with symptoms of abdominal pain and diarrhea, is a specific subtype of BD that accounts for approximately 3-16% of BD cases [2].

Conventionally pharmacological treatments include 5-aminosalicylic acid (5-ASA), systemic corticosteroids, and immunosuppressive agents such as thiopurines [3-5]. However, many patients fail to respond to these treatments [6, 7]. The clinical outcomes for these agents in the treatment of intestinal BD are thus limited. In recent years, accumulating evidence on the efficacy of infliximab (IFX), an anti-tumor necrosis factor (TNF)-α agent, in the management of Behcet’s uveitis [8], has encouraged the use of anti-TNF-α agents for management of intestinal BD [9]. Very recently, adalimumab (ADA) has been demonstrated to be effective and safe for patients with intestinal BD [10]. As a consequence, ADA was approved for intestinal BD in Japan in 2013. However, in the clinical setting, little information is currently available regarding the long-term efficacy of ADA against intestinal BD [11].

This study retrospectively assessed the 52-week efficacy of ADA for intestinal BD refractory to conventional treatments including 5-ASA, corticosteroids, and thiopurines.

## Case Report

### Patients

Between October 2010 and July 2015, eight consecutive patients were referred to Nagoya City University Hospital for the treatment of intestinal BD. Patients were diagnosed with BD according to diagnostic criteria defined by the BD Research Committee of Japan [12]. Briefly, BD that includes four major features (recurrent oral ulceration, genital ulceration, ocular lesion, and typical skin lesion) is classified as complete-type BD, while BD with three major features, or two major and two minor features (arthritis without deformity and ankylosis, ileocecal ulcers, epididymitis, vascular lesions or central nervous system symptoms), or typical ocular lesion plus one major or two minor features is classified as incomplete-type BD, and BD with one major feature is classified as suspected BD.

### Treatments and assessments

The eight patients, including one with postoperative recurrence, were given ADA 160 mg in week 0, 80 mg in week 2, and 40 mg every other week over 52 weeks for refractory BD that failed to respond to conventional treatments including mesalazine (MLZ) or prednisolone (PSL), and azathioprine (AZA). Global gastrointestinal (GI) symptom score and endoscopic assessment were performed as previously reported [10]. The primary outcome was the percentage of patients showing marked improvement (MI) (score 0 or 1 for both global GI symptoms and endoscopic assessments) at 10 and 52 weeks after starting ADA, and secondary outcomes were the percentage of clinical remission (score 0 for both global GI symptoms and endoscopic assessments). Any adverse event was recorded, along with date of onset, severity, outcome, and relationship of event to these therapies. Data are presented as mean value. Missing data were imputed using last observation carried forward (LOCF) [10]. Data are presented as means ± standard error of the mean, and comparisons were made using a paired *t*-test. A significance level of 0.05 was used for all statistical tests, and two-tailed tests were applied when appropriate.

**Figure 1:**
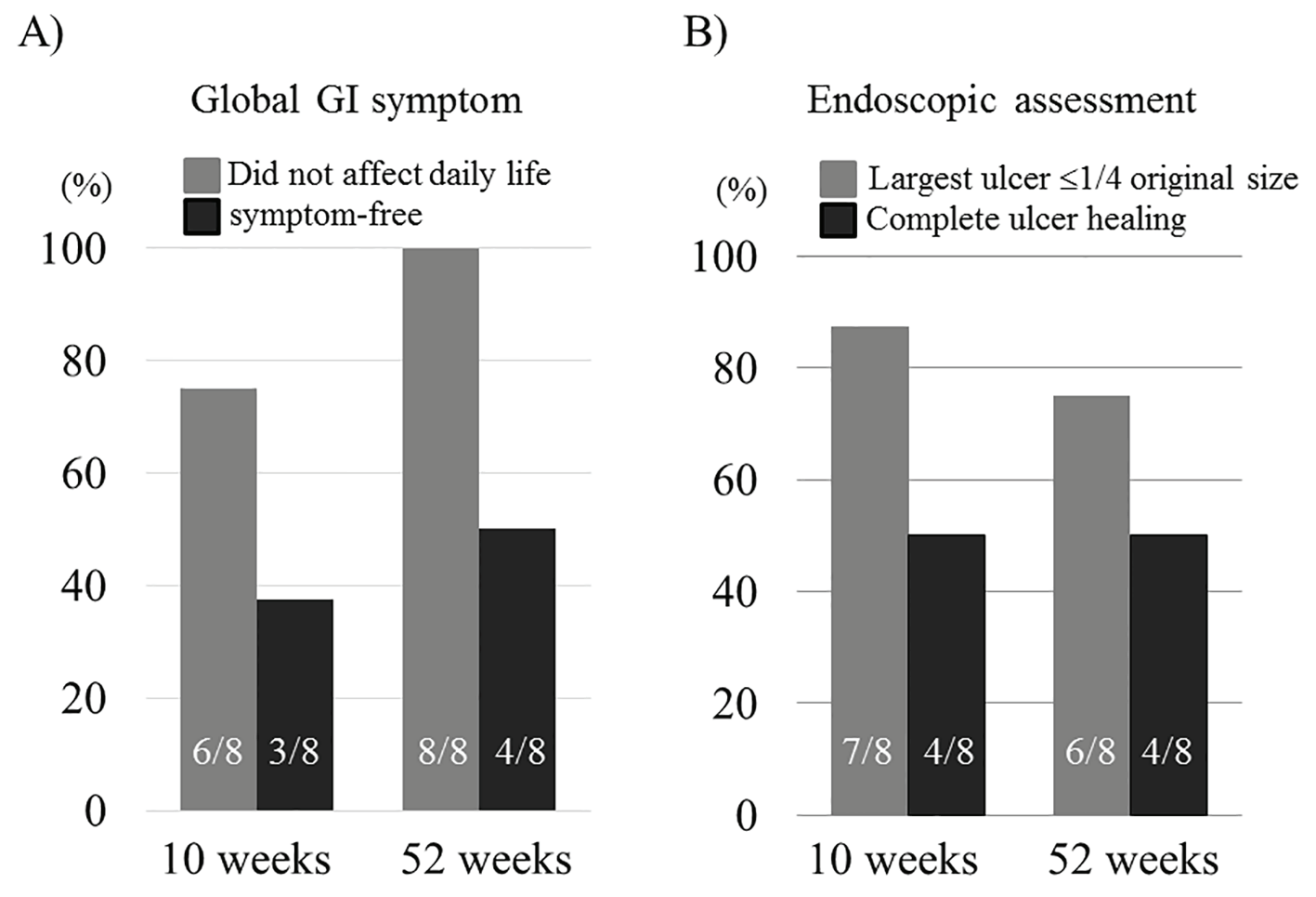
Evolution of the percentage of global GI symptom score 0 or ≤ 1 (A) and endoscopic assessment score 0 or ≤ 1 (B) at 10 and 52 weeks in the eight cases receiving ADA. Data are presented as mean ± SEM.

The demographic data are shown in Table 1. Mean age was 46.6 years. Disease type included seven incomplete cases and one suspected case. Ulcer size at the ileocecal valve included two cases with ulcers of 1 - 2 cm, three cases of 2 - 3 cm, and three cases of ≥ 3 cm. Concurrent medications included MLZ, PSL, and AZA. Of the eight patients receiving ADA (160/80/40 mg every other week), five patients (62.5%) achieved MI at 10 weeks, and six (75%) patients showed MI at 52 weeks. In addition, complete remission was obtained in two patients (25%) at both 10 and 52 weeks. Repeated oral aphtha persisted in three patients without other major features after starting ADA (Table 2). Improvement of global GI symptoms to a score of ≤ 1 was seen in six patients (75%) at 10 weeks and eight patients (100%) at 52 weeks. Furthermore, improvement to score 0 was seen in three patients (37.5%) at 10 weeks and four patients (50%) at 52 weeks, respectively (Fig. 1A). Moreover, improvement of endoscopic assessment to a score of ≤ 1 was also seen with seven patients (87.5%) at 10 weeks and six patients (75%) at 52 weeks, respectively. A score of 0 was seen in four patients (50%) at both 10 and 52 weeks (Fig. 1B).

**Table 1:** Baseline Demographic Variables of the Eight Patients With Intestinal BD Refractory to Conventional Medications Who Were Given ADA

Demography	Number (%) (N = 8)
Male sex	4 (50%)
Mean age	46.6
Smoking (current)	1 (12.5%)
Alcohol	1 (12.5%)
Disease type	
Complete/incomplete/suspicious	0/7/1
GI symptom score	
3	5 (62.5%)
4	3 (37.5%)
Ulcer size at ileocecum	
1 - 2 cm	2 (25%)
2 - 3 cm	3 (37.5%)
≥ 3 cm	3 (37.5%)
Concomitant drugs	
Mesalazine	4 (50%)
Prednisolone	4 (50%)
Azathioprine	1 (12.5%)

**Table 2:** Clinical Course up to 52 Weeks

Case No.	Age	Sex	Type	Postoperative recurrence	0 week					Treated ADA	10 weeks				Marked improvement	52 weeks				Marked improvement
					GI symp	Ulcer size	Oral aphtha	CRP	Pretreated		GI symp	End score	CRP	Oral aphtha		GI symp	End score	CRP	Oral aphtha	
1	65	M	Incomplete	-	3	3 cm	+	5.44	PSL (5)	+	1	1	0.37	-	+	0	2	0.43	-	-
2	29	F	Incomplete	-	4	1 - 2 cm	+	0.18	PSL (5)	+	2	0	0.10	-	-	1	0	0.14	-	+
3	39	M	Incomplete	-	4	3 cm	+	0.38	PSL (10)	+	2	0	0.05	-	-	1	0	0.05	-	+
4	41	F	Suspicious	-	4	2 - 3 cm	+	0.20	PSL (failed), MLZ (2,000)	+	0	1	0.05	-	+	0	1	0.03	-	+
5	68	M	Incomplete	+	3	2 - 3 cm	+	0.33	PSL (25), AZA (25)	+	1	1	0.08	-	+	1	1	0.15	-	+
6	59	M	Incomplete	-	3	1 - 2 cm	+	0.42	MLZ (3,000)	+	0	0	0.04	+	+	0	0*	0.03	+	+
7	15	F	Incomplete	-	3	2 - 3 cm	+	0.58	MLZ (2,000)	+	0	0	0.04	+	+	0	0	0.07	+	+
8	57	F	Incomplete	-	3	3 cm	+	0.34	MLZ (intolerance)	+	1	2	0.65	+	-	1	2	0.37	+	-

Numbers in parentheses indicate doses (mg/day) of concurrent drugs. Asterisk means last observation carried forward because endoscopic score of patient 6 presented with the 32-week score. MLZ: mesalazine; PSL: prednisolone; AZA: azathioprine; CRP: C-reactive protein; ADA: adalimumab; symp: symptom; End score: endoscopic score; failed: failed to achieve clinical response.

Mean C-reactive protein (CRP) level in eight patients receiving ADA was 0.98 ± 0.64 mg/dL at baseline. This decreased to 0.17 ± 0.08 mg/dL at 10 weeks and 0.16 ± 0.05 mg/dL at 52 weeks, showing no significant difference (Table 2). All patients taking PSL were able to taper off by 10 weeks after starting ADA. No adverse events were observed in any patients during the 52 weeks. ADA therapy was therefore considered safe and well tolerated.

## Discussion

We have reported herein the efficacy of ADA induction and maintenance in eight consecutive cases with intestinal BD.

Regarding anti-TNF-α antibodies for intestinal BD, a retrospective clinical cohort study investigating the efficacy of IFX in 43 patients with refractory intestinal BD showed that 12 patients (80%) responded to IFX, and that eight patients (53%) were in remission with no GI symptoms and normal CRP level at 10 weeks. In addition, response to IFX was maintained in seven of the 11 patients (64%) receiving maintenance therapy for 12 months. However, the cumulative recurrence rates at 12 and 24 months were 29% and 51%, respectively, and the cumulative surgery rates at 12 and 24 months were 13% and 37%, respectively [13]. The first prospective clinical trial investigating the efficacy of ADA in 20 patients with refractory intestinal BD showed that eight patients (40%) and 12 patients (60%) achieved MI in week 8 - 12 and week 52, respectively. In addition, three patients (15%) and four patients (20%) achieved complete remission in week 8 - 12 and week 52, respectively. These suggest that in clinical settings, anti-TNF-α antibodies such as IFX and ADA are efficacious therapies to induce and maintain clinical improvement and remission in patients with intestinal BD. Moreover, these effects are supported by the pathogenesis of BD, in which increased expression of TNF-α by γδT cells and monocytes in peripheral blood has been confirmed in patients with active BD [14, 15], and increased levels of TNF-α mRNA have been detected in ulcer lesions, which decrease after treatment [16].

In addition, of the five patients receiving PSL, PSL could be withdrawn at 10 weeks in all patients (100%) (Table 2). The clinical trial of ADA for the treatments of immune-mediated diseases such as Crohn’s disease and intestinal BD demonstrated that 29% of Crohn’s disease patients at 56 weeks [17] and 61.5% of intestinal BD patients at 52 weeks [10] taking corticosteroids at baseline who were treated with the ADA 40 mg every other week maintenance therapy could discontinue corticosteroids, respectively. These suggest that ADA treatment is useful to taper off corticosteroids.

However, patients with intestinal BD often fail to respond to anti-TNF-α antibodies [13]. In addition, even if dose-escalation of ADA to 80 mg every other week was tried and an increased trough level of serum ADA is maintained in patients with inadequate response or disease flare, some patients fail to achieve MI and clinical remission [10]. ADA monotherapy for refractory intestinal BD thus has limitations.

In conclusion, ADA is an effective, well-tolerated treatment of intestinal BD in patients who are refractory to conventional therapy.

## Abbreviations

BD: Behcet’s disease
ADA: adalimumab
5-ASA: 5-aminosalicylic acid
TNF: tumor necrosis factor
IFX: infliximab
MLZ: mesalazine
PSL: prednisolone
AZA: azathioprine
GI: gastrointestinal
MI: marked improvement
LOCF: last observation carried forward
CRP: C-reactive protein
